# Re-Hospitalization in First Six Months After Live Related Renal Transplantation: Risk Factors, Burden, Causes and Outcomes

**DOI:** 10.7759/cureus.22043

**Published:** 2022-02-09

**Authors:** Sommiya Dashti, Murtaza Dhrolia, Kiran Nasir, Ruqaya Qureshi, Aasim Ahmad

**Affiliations:** 1 Nephrology, The Kidney Centre Post Graduate Training Institute, Karachi, PAK

**Keywords:** infection, rejection, outcome, causes, risk factors, live-related renal transplantation, re-hospitalization

## Abstract

Objective

The aim of our study was to evaluate the incidence, causes, risk factors, outcomes, and cost of hospital readmission after live related renal transplantation (LRRT).

Methods

We conducted a cross-sectional study and followed patients’ re-admissions for six months whose LRRT was done in our center between September 2019 and June 2020.

Results

We recruited 53 patients, 40 (75.5%) were male. The mean age was 36.9 ± 11.9 years. Donor gender was similar, and their mean age was 31.6 ± 9.2 years. The mean length of hospital stay after LRRT was 14 ± 2.2 days. A total of 81.1% were readmitted after LRRT within the first six months, with a total of 113 readmissions. The median time of readmission after LRRT was 66 days. The median readmission hospital stay was four days. The causes of readmission were surgical in 11 (9.7%), medical in 89 (78.8%), and combined medical and surgical in 13 (11.5%). Infection was the most common medical cause, followed by rejection. Statistically significant difference between readmission and non-readmission groups was found in estimated glomerular filtration rate (eGFR) at six month 61.3 ± 25.9 vs. 84.3 ± 36.1 mL/min/1.73 m^2^ respectively (p = 0.02). The median cost of readmission was PKR 40629, equivalent to USD 261.

Conclusion

Over three-fourths of the patients were readmitted after LRRT within the first six months. The most common causes were infection and rejection. Readmissions after LRRT are associated with lower graft function at six months and a significant cost burden on the health system.

## Introduction

Since the first renal transplant by Murray in 1954 [1], it is now considered the treatment of choice for patients with end-stage kidney disease (ESKD). It offers improvements in life expectancy and quality of life for most patients compared to remaining on dialysis [2-3].

Re-hospitalization after renal transplant surgery is common [4-5], because of the complexity of immunosuppression regimens, rejection, infection, and other transplant-specific complications and has been shown to have a significant impact on the health care cost, morbidity, graft loss, and death [4,6-7].

After transplantation, within the first 30 days, surgical complications related to the procedure are predominant [4, 8]. After that, immunological and infective causes tend to be more common. Early acute rejection (EAR) occurs in less than three months and late acute rejection (LAR) occurs after three months of transplant [9]. The majority of the infections are seen within 1-6 months [10] and found to be a significant problem in developing countries post-transplant [11].

Examining cause-specific re-hospitalization in these renal transplant patients may help identify possible avoidable factors that may prevent readmission. There is a lack of data about hospital readmission after kidney transplantation from developing countries. Pakistan is a developing country where more than 40% of the population live below the poverty line, transplant programs are mainly based on community-government partnerships, and local philanthropy plays a vital role [12]. Donation of organs from non-relatives is prohibited as per country law [13] in an attempt to combat organ trafficking. These cultural, social, and economic difficulties result in reduced transplantation activity, with re-hospitalization further burdening these patients, particularly in patients that pay for transplants themselves.

In our study, we aimed to evaluate the incidence, causes, risk factors, outcomes, and cost of hospital readmission after live related renal transplantation (LRRT) in post-transplant patients of our centre, which to the best of our knowledge, is the first study from private-sector transplant program from Pakistan.

## Materials and methods

In this observational, cross-sectional study, we followed all the re-admissions of kidney recipients for six months after LRRT whose renal transplant was done at our centre between September 2019 and June 2020. We excluded multi-organ transplant patients, whose transplants were done outside our hospital and unrelated renal transplants and those who lost graft during admission for LRRT or were lost to follow up. Day-care admission for Double-J (DJ) removal was also not included. Inform consent was waived as data was collected without patient interaction. Transplant procedures were conducted using a standard surgical technique, and all patients had a Double-J stent placed.

Re-hospitalization was defined as a hospital admission that occurred for any reason after discharge from the initial transplant hospitalization. The patients were followed for age, gender, comorbidity, cause of the end-stage kidney disease (ESKD), donor age and gender, human leukocyte antigens (HLA) matching, induction therapy, maintenance immunosuppressant, graft function, other laboratory parameters at first discharge after renal transplantation, length of initial hospital stay during renal transplantation, day of initial hospital discharge after renal transplantation (weekday vs. weekend), the time interval between transplantation and admission, length of hospital stay, day of admission (weekday vs. weekend), causes of admission, outcome and admission cost.

Causes of admission were categorized broadly into surgical and medical. Medical causes were further sub-classified into infection, rejection, calcineurin inhibitor (CNI) toxicity, and miscellaneous. As the admissions are possible for more than one cause, the frequencies of causes reported are more than the number of admissions. The outcomes in relation to estimated glomerular filtration rate (eGFR), graft loss, and death at six months of LRRT were compared between readmission and non-readmission groups.

The admission cost was taken as the total direct costs that the hospital charged for re-hospitalization of the patient (this included the costs of hospital stay, medications, surgical procedures, laboratory, imaging tests, and miscellaneous costs). The cost was adjusted for the inflation rates at 5% per year and converted from Pakistani rupees (PKR) to United States Dollar (USD) as of the conversion rate for the year of admission.

The data was entered and analyzed on SPSS version 21 (IBM Corp., Armonk, NY). Mean ± STD and median with interquartile range were computed for continuous variables, while the frequencies with percentages were calculated for categorical variables. Association between categorical variables was established by Chi-square test or Fisher exact test; on the other hand, independent t-test was applied for normally distributed continuous data, while Mann-Whitney U test was executed for skewed variables. Shapiro Wilk’s test checked the normality of data. A P-value of ≤0.05 was set as a significant level.

## Results

We recruited 53 patients for this study, of which 40 (75.5%) were male, while females were 13 (24.5%). The mean age of patients was 36.9 ± 11.9 years. The most common comorbidity was hypertension (HTN) (50, 94.3%) in these patients, while unknown kidney disease (bilateral small shrunken kidneys) was the most prevalent cause of chronic kidney disease (CKD) (28, 52.8%). The mean CKD vintage of patients before LRRT was 4.0 ± 5.0 years, while the patients remained on hemodialysis (HD) before LRRT for 1.0 ± 1.2 years. Most patients, 44 (83%), were being dialyzed via arterio-venous fistula (AVF) at the time of LRRT.

An almost equal number of recipients received kidneys from males (49.1%) and females (50.9%) donors. Twenty-three (43.4%) were from siblings. The mean age of the donor was 31.6 ± 9.2 years. The majority of our patients had ≥3 antigen HLA match (40, 75.5%), and standard induction (with methylprednisolone) was given to most of the patients (42, 79.2%), with 49 (92.5%) were started on maintenance immunosuppression with prednisolone, cyclosporine, and mycophenolate mofetil (MMF). The mean length of initial hospital stay after renal transplantation surgery was 14 ± 2.2 days. During the initial hospital stay after LRRT, five (9.4%) patients developed surgical complications while 31 (58.5%) suffered medical problems. One patient required hemodialysis after transplant surgery. Twenty-three (43.4%) underwent transplanted kidney biopsy, and 21 (39.6%) had renal allograft rejection episodes. The mean best creatinine achieved after LRRT was 1.27 ± 0.89 mg/dl while creatinine at discharge after LRRT surgery was 1.47 ± 1.03 mg/dl. Most of the patients, 46 (86.6%), were discharged on working days after transplant surgery. Demographic, clinical, and laboratory characteristics among LRRT patients and their comparison in readmission and non-readmission groups are presented in Table 1.

**Table 1:** Baseline characteristics among LRRT patients in readmission and non-readmission group LRRT: Live related renal transplantation; CKD: Chronic kidney disease; HD: Hemodialysis; ADPKD: Autosomal dominant polycystic kidney disease; HLA: Human leukocyte antigens; IQR: Interquartile range.

Variable	Total (n=53)			Readmission group n=43 (81.1%)			Non-readmission group n=10 (18.9%)			p-value
	Mean±STD	Median	IQR	Mean±STD	Median	IQR	Mean±STD	Median	IQR	
Age	36.9±11.9	36	19	36.4±12	34	20	38.9±12.2	36.5	24	0.585
Duration of CKD	4.0±5.0	2	4	4.3±5.1	2	5	3.1±5	1.5	2.2	0.292
HD vintage	1.0±1.2	0.8	0.7	1±1.3	0.8	0.7	1±1.1	1.2	1.3	0.908
Donor Age	31.6±9.2	28	12	31.8±8.8	29	11	30.8±11.2	27.5	20	0.682
Hospital stay during LRRT	14±2.25	13	1	13.9±2.3	13	1	14.4±2.1	13.5	4	0.427
Best Creatinine after LRRT	1.27±0.9	1	0.6	1.2±0.8	1	0.6	1.5±1.3	1	1.2	0.785
Male	40 (75.3)			36 (83.7)			4 (40)			0.004
Female	13 (24.5)			7 (16.3)			6 (60)			
Comorbidity: Hypertension	50 (94.3)			40 (93)			10 (100)			0.615
Diabetes Mellitus	4 (5.7)			3 (7)			1 (10)			0.999
Hepatitis B	2 (3.8)			2 (4.7)			0			0.999
Hepatitis C	5 (9.4)			3 (7)			2 (20)			0.235
Causes of CKD: Unknown	28 (52.8)			21 (48.8)			7 (70)			0.734
Diabetic kidney disease	1 (1.9)			1 (2.3)			0			
Glomerulonephritis	12 (22.6)			10 (23.3)			2 (20)			
Renal stone	8 (15.1)			7 (16.3)			1 (10)			
ADPKD	4 (7.5)			4 (9.3)			0			
HD-access: Arteriovenous fistula	43 (81.1)			35 (81.4)			8 (80)			0.919
Temporary Catheter	10 (18.9)			8 (18.6)			2 (20)			
Donor Gender: Male	26 (49.1)			23 (53.5)			3 (30)			0.181
Female	27 (50.9)			20 (46.5)			7 (70)			
HLA-matching: ≥3 antigen	40 (75.5)			34 (79.1)			6 (60)			0.207
<3 antigen	13 (24.5)			9 (20.9)			4 (40)			
Induction Therapy: Methylprednisolone	42 (79.2)			36 (83.7)			6 (60)			0.078
Basiliximab	10 (18.9)			7 (16.3)			3 (30)			
Rituximab+plasmapheresis	1 (1.9)			0			1 (10)			
Complications of LRRT: No	17 (32.1)			14 (32.6)			3 (30)			0.999
Surgical	5 (9.4)			4 (9.3)			1 (10)			
Medical	31 (58.5)			25 (58.1)			6 (60)			
Episodes of rejection after LRRT: No	32 (60.4)			26 (60.5)			6 (60)			0.978
Yes	21 (39.6)			17 (39.5)			4 (40)			
Day of discharge after LRRT: Weekday	46 (86.8)			37 (86)			9 (90)			0.739
Weekend	7 (13.2)			6 (14)			1 (10)			

Baseline laboratory parameters are discussed in Table 2.

**Table 2:** Baseline laboratory parameters at initial discharge among LRRT patients in readmission and non-readmission group LRRT: Live related renal transplantation; IQR: Interquartile range.

Variable	Total (n=53)			Readmission group n=43 (81.1%)			Non-readmission group n=10 (18.9%)			p-value
	Mean±STD	Median	IQR	Mean±STD	Median	IQR	Mean±STD	Median	IQR	
Creatinine	1.5±1	1.2	0.7	1.4±0.8	1.3	0.7	1.8±1.8	1.1	1.4	0.585
Hemoglobin	10±1.4	9.8	1.9	10.2±1.4	10.2	2.1	9.3±1.3	9.3	2	0.071
Leukocyte	11.5±5.3	11.1	6	11.1±5.5	10.4	6.1	12.8±4.6	12.3	4.6	0.203
Platelet	250±134	225	93	256±142	225	90	227.3±70	230	112	0.733
Albumin	2.9±0.5	2.9	0.6	3±0.4	2.9	0.7	2.6±0.5	2.7	0.4	0.031
Sodium	137.5±3.8	137	6	137.3±3.8	137	6	138.1±3.5	138	4.3	0.563
Potassium	4.3±0.6	4.1	0.7	4.3±0.6	4.1	0.7	4.3±0.5	1.3	0.8	0.978
Bicarbonate	21.3±3.3	21	4	20.9±3.2	21	4	23.2±3.5	22	4.5	0.123
Calcium	8.9±0.8	9	1.2	8.9±0.7	8.8	1.1	9.2±1	9.3	1.1	0.313
Phosphorus	2.5±1.5	2.2	1.1	2.4±1.2	2.3	0.9	2.8±2.5	1.9	1.5	0.317
Magnesium	1.58±0.44	1.5	0.47	1.55±0.4	1.47	0.4	1.75±0.5	1.7	0.7	0.213
Alanine-aminotransferase	21.7±23.4	14	14	22±23.4	16	14	20.7±24.4	12	18.3	0.187
Aspartate aminotransferase	18.5±8.7	16	11	18±8.3	16	11	20.7±10.3	17.5	15.5	0.432
Cyclosporin level C0	204±105	181	151	207±100	182	142	188±129	141	204	0.345
Cyclosporin level C2	1094±390	1057	515	1068±359	1057	481	1206±509	1226	792	0.348

Out of 53 patients, 43 (81.1%) patients were readmitted after LRRT within the first six months, with 23 (43.4%) admitted within 30 days and 33 (62.5%) within three months. Forty-three transplanted patients resulted in a total of 113 readmissions within six months of renal transplantation. The average admission days per patient was 2.1 ± 1.89, 18 (34%) had more than two admissions. The median time of readmission after LRRT was 66 days, with 32 (28.3%) readmissions were within 30 days, 41 (36.3%) readmissions were from >30 days to three months, and 40 (35.4%) were from three months onward. Eighty-six (76.1%) admissions were made on weekdays and 27 (23.9%) on weekends. The median readmission hospital stay was four days.

There was no impact of demographic, clinical, and laboratory variables on readmission, that was statistically significant except for the patient’s gender. Males (36, 90%) were readmitted more than females (7, 53.8%) (p = 0.004), while serum albumin at initial discharge after transplant surgery was found to be better in readmission group (p = 0.03) (Table 1).

The causes of readmission were isolated surgical in 11 (9.7%), medical in 89 (78.8%), and combined medical and surgical in 13 (11.5%). Thirteen (54.2%) out of 24 surgical readmissions causes and 74.5% of readmissions due to medical causes occurred within 30 days after discharge. The most common surgical cause of admission was ureter-related complications (7, 29%) followed by lymphocele (6, 25%). Ninety-seven (95%) out of 102 admissions due to medical causes had a single medical cause, while five (4.9%) admission had multiple medical reasons. Infection was the most common medical cause and was found in 50 (49%), followed by rejection (37, 36%), miscellaneous (15, 14.7%), and CNI toxicity (6, 5.9%). The most common infective cause was urinary tract infection (UTI) found in 19 (38%). Twenty-two (59.5%) of rejection episodes were early acute rejection (EAR), while 15 (40.5%) were late acute rejection (LAR). Table 3 shows the distribution of causes of readmission in the first six months of LRRT.

**Table 3:** All causes of hospital readmission in the first six months after LRRT LRRT: Live related renal transplantation

Causes	n (%)
Surgical	24 (19)
Ureteric related	07 (29)
Urine leak	04 (3.5)
Ureteric obstruction	03 (2.7)
Lymphocele	06 (25)
Wound Infection	03 (12.5)
Miscellaneous	08 (33)
Pancreatic pseudocyst	02 (1.8)
Pancreatitis	01 (0.9)
Paralytic ileus	01 (0.9)
Urinary retention	01 (0.9)
Perigraft hematoma	01 (0.9)
Native Kidney Nephrectomy	01 (0.9)
Hemorrhoidectomy	01 (0.9)
Medical	102 (81)
Infection	50 (49)
Urinary tract infection	19 (16.8)
Septicemia	14 (12.4)
Gastroenteritis	09 (8.0)
Cytomegalovirus	04 (3.5)
BK virus	01 (0.9)
Tuberculosis	01 (0.9)
Unknown	02 (1.8)
Rejection	37 (36.3)
Early acute rejection	22 (19.5)
Late acute rejection	15 (13.3)
Calcineurin inhibitor toxicity	06 (5.9)
Miscellaneous	15 (14.7)
Volume depletion	10 (8.8)
Acute tubular necrosis	03 (2.7)
Hyperglycemia	01 (0.9)
Anemia	01 (0.9)

Comparing the outcomes between readmission and non-readmission groups, a statistically significant difference was found in eGFR at six months (61.3 ± 25.9 vs. 84.3 ± 36.1 mL/min/1.73 m^2^ respectively) (p = 0.02). When we further compared eGFR within the readmission group, those with more than two admissions were found to have decreased eGFR than those with less than or equal to two admissions (55.1 ± 26.5 vs. 65.7 ± 25.1) (p = 0.036). No statistically significant difference was found in graft loss and death between the two groups (Table 4).

**Table 4:** Outcome between readmission and non-readmission groups at six months of LRRT LRRT: Live related renal transplantation; eGFR: Estimated glomerular filtration rate; IQR: Interquartile range.

Variable	Total (n=53)			Readmission group n=43 (81.1%)			Non-readmission group n=10 (18.9%)			p-value
	Mean±STD	Median	IQR	Mean±STD	Median	IQR	Mean±STD	Median	IQR	
Serum creatinine	1.7±1.4	1.38	0.6	1.7±1.2	1.5	0.6	1.7±2.2	1.1	0.7	0.042
eGFR	65.6±29.1	63	34	61.3±25.9	59	32	84.3±36.1	93	39	0.023
Graft loss	4 (7.5%)			3 (7%)			1 (10)			0.744
Death	2 (3.8%)			2 (4.7%)			0			0.487

The overall graft survival rate of our LRRT patients was 92.5% at six months, with 93% in the readmission group and 90% in the non-readmission group. Overall, the patient survival rate was 96% at six months, with 95% in readmission and 100% in the non-readmission group. The mean cost of readmission was PKR 61542 ± 62086 (Median 40629), equivalent to USD 396 ± 402 (Median 261, @ 1 USD = 155.6).

## Discussion

Economic constraints, with insufficient health spending and cultural and societal hurdles in Pakistan, resulted in lower transplantation activity [11]. Re-hospitalization after transplant further burdens these patients with a significant impact on the health care cost, morbidity, graft loss, and death [4, 6-7]. There is a lack of data on the incidence, causes, risk factors, outcomes, and cost of hospital readmission after kidney transplantation (KT) from developing countries. Our study aimed to fill that gap and get insight into the quality measurement of live-related renal transplants at our centre. This is the first study from the private-sector transplant program in Pakistan.

In our study, 81.1% of patients were readmitted after LRRT within the first six months, with 43.4% admitted within 30 days and 62.5% within three months. A study from China [14] showed a readmission rate of 22.59% within one year after KT while data from the United States Renal Data System (USRDS) showed 31% (range from 18% to 47% among different transplant centres) of recipients readmitted within 30 days of discharge after KT [8]. A study from Canada [15] found early readmission rates of 19.4% at 30 days and 26.8% at 90 days post-transplant. Our high readmission rate is surprising and demands comprehensive adjudication between potentially avoidable factors, the process of care, and unpredictable acceptable risk due to the uniquely vulnerable nature of transplant procedure in the face of limited resource setup. A high re-admissions rate at our centre needs to be further evaluated.

The causes of readmission were found to be multifactorial. The most common causes were infection and rejection. Infection was found in 44% of overall admissions within six months. The proportion of readmissions attributable to infections was equally distributed within 30 days, >30 to three months, and three months onward. Our readmission rate is much higher than the infection-related readmissions found in the United States Renal Data System (12%) [8] and Canada (21% within 30 days and 28.1% within 90 days) [15]. However, it is similar to the findings of studies from Iran (51% and 49.6%) [5, 16]. The tendency of increased infectious events after transplant has been attributed to the use of immunosuppressive drugs, exposure to nosocomial pathogens, and the need for invasive intravascular and urinary devices.

The most common infection found in our study was UTI, which correlates with studies from Canada [15], Iran [16], and Colombia [17]. In addition, a study from our centre found that 33% of renal transplant recipients had at least one episode of urinary tract infection, and the structural abnormalities, renal stone disease, delay in removing Foley's catheter, and increased post-transplant hospital stay are risk factors leading to UTI in renal transplant recipients [18].

In contrast to the studies from Pakistan [19] and India [20], which showed 12-20% incidence of tuberculosis in developing countries, we found tuberculosis in only one patient. Moreover, in our centre, none of the transplant recipients received isoniazid prophylaxis. Since tuberculosis incidence was found higher after two years of transplantation period [19], explaining the absence of frequent tuberculosis in the early post-transplantation period studied in our study.

Rejection episodes in our study were early acute rejection (EAR) in 59.5% while 40.5% were late acute rejection (LAR). Rejection has also been found to be a common reason for readmission in several other studies of post-transplant early hospital readmissions [5, 15].

The overall incidence of surgical complications was low within the six months after LRRT, which correlates with the study from Famure et al. [15]. However, a majority (54.2%) occurred within 30 days, similar to what is reported by McAdams et al. [8]. A study from Iran found surgical complications as the most frequent reason for readmission within the first six months [5].

When we compared the impact of demographic, clinical, and laboratory variables of patients at the time of LRRT on the readmission rate, we did not find any statistically significant association between already identified significant factors from literature in our study, except for the patients' gender. Males were readmitted more than females in our study. Contrary to this, McAdams et al. [8] found that women with diabetes had a higher risk of 30 days’ readmission than men with diabetes. Factors at the time of transplant that were found to be associated with the increased hospital readmission in different studies [8, 14, 15, 21-23] are older age, higher body mass index (BMI), diabetes mellitus, a history of heart disease or chronic obstructive pulmonary disease, expanded criteria deceased donor (vs. living donor), use of an induction agent, any degree of human leukocyte antigens matches, longer mean length of stay (LOS) for the transplant hospitalization, being discharged on the weekend, frailty, median time on dialysis, being transplanted pre-emptively rather than having had conventional hemodialysis, and dialysis modality. However, these associations were not reproduced in the following studies. Chu et al. [14] did not find an association between age, gender, BMI or immunosuppressive induction with readmissions but found dialysis modality as a risk factor for readmission while Famure et al. [15] found recipient history of chronic lung disease, median time on dialysis, being transplanted pre-emptively rather than having had conventional hemodialysis as the factors associated with elevated risks of readmission within both 30- and 90-day post-transplant. Increased length of stay for the transplant hospitalization was found to be a risk factor for readmission in many studies [8, 14, 15], however not found as a risk factor in the study by Taber et al. [21].

When we compared outcomes between readmission and non-readmission groups, a statistically significant (p = 0.02) difference was found in eGFR at six months. When we further compared eGFR within the readmission group, there was a statically significant (p = 0.036) reduction in eGFR with those with multiple readmissions. McAdams et al. [7] found that patients readmitted within 30 days of transplant discharge were more likely to experience kidney allograft failure and mortality. Luan et al. [6] found that only patients readmitted to the hospital two or more times experienced higher rates of death or graft failure. In a study by Harhay et al. [22], early hospital readmission was associated with an increased risk of mortality but not all-cause graft loss. Overall graft and patient survival rate of our LRRT patients was 92.5% and 96% at six months, respectively. Another single-centre study from Pakistan found one-year graft survival of 92% and one-year patient survival of 94% [11].

The mean cost of readmission was PKR 61542 ± 62086 (Median 40629) equivalent to USD 396 ± 402 (Median 261, @ 1 USD = 155.6). This cost is lower compared to hospitalization charges during the first six months mentioned in a study from Iran (709 ± 366 USD). However, it is still a matter of concern for the country where 33% of people live below the poverty line with only $1 a day for sustenance [11].

Our study was a single-centre study with a small sample size. Despite this, our study serves as baseline data for future studies on this subject, especially in this part of the world. High incidence of readmission rate after LRRT with its significant effect on graft function and health care cost demands understanding and prevention of potentially avoidable factors and measures to improve quality of care. Studies that examined the potentially avoidable hospital readmission after transplant found variable results. Harhay et al. [22] found that only 9% of early readmissions were preventable, while Goldfield et al. [23] found that 21% are preventable.

In contrast to developed countries, developing countries like Pakistan confront economic limitations and essential public health fundamentals such as clean water, sanitation, and vaccination. It also faces the technical challenges of surgery, increased rate of infections, and limited medical, surgical, and nursing work forces with the required expertise. These factors probably give a higher rate of preventable readmission in our patients if the study identifies potentially avoidable factors in this cohort. If a sufficient number of hospital re-admissions are preventable, interventions may be designed and tested to decrease the burden of re-admissions.

The limitation of the study is that the sample size was small, so for these types of studies, we need larger prospective studies in collaboration with different institutes.

## Conclusions

In our study, over three-fourths of the patients were readmitted after LRRT within the first six months. This rate of readmissions is higher than studies from developed countries. The causes of readmission were found to be multifactorial. The most common causes were infection and rejection. There has been substantial variability in the specific risk factors identified in various studies. However, we did not find any statistically significant association between already identified significant factors from literature in our study, except for males that were readmitted more than females. Readmissions after LRRT are associated with lower graft function at six months and a significant cost burden on the health system.
